# A Retrospective Evaluation of the Cardiometabolic Profile of Patients with COPD-Related Type 2 Respiratory Failure in the Intensive Care Unit

**DOI:** 10.3390/medicina61040705

**Published:** 2025-04-11

**Authors:** Oral Mentes, Deniz Celik, Murat Yildiz, Kerem Ensarioglu, Mustafa Ozgur Cirik, Tulay Tuncer Peker, Fatma Canbay, Guler Eraslan Doganay, Abdullah Kahraman

**Affiliations:** 1 Department of Intensive Care, Gulhane Training and Research Hospital, 06010 Ankara, Turkey; omentes@live.com (O.M.); ttuncerpeker@gmail.com (T.T.P.); 2 Department of Pulmonary Medicine, Faculty of Medicine, Alanya Alaaddin Keykubat University, 07425 Antalya, Turkey; 3 Department of Pulmonary Medicine, Atatürk Sanatorium Research Hospital, Faculty of Medicine, Ankara Turkey Health Sciences University, 06830 Ankara, Turkey; drmuratyildiz85@gmail.com (M.Y.); kerem.ensarioglu@gmail.com (K.E.); 4 Department of Anesthesiology, Atatürk Sanatorium Research Hospital, Faculty of Medicine, Turkey Health Sciences University, 06100 Ankara, Turkey; dr.ozgurr@hotmail.com (M.O.C.); gulerdoganay@hotmail.com (G.E.D.); 5 Department of Pulmonary Medicine, Dr. Burhanettin Nalbantoğlu Public Hospital, Lefkoşa 99010, Cyprus; canbayfatma5@gmail.com; 6 Department of Intensive Care, Etlik City Hospital, 06170 Ankara, Turkey; abdullahhero100@gmail.com

**Keywords:** COPD, type 2 respiratory failure, metabolic profile, intensive care unit, comorbidities, obesity

## Abstract

*Background and Objectives*: Chronic obstructive pulmonary disease (COPD) is a notable cause of morbidity and mortality worldwide and can become complicated by Type 2 respiratory failure. This study aimed to analyze the cardiological and metabolic comorbidities of patients admitted to the intensive care unit (ICU) due to COPD-related Type 2 respiratory failure and evaluate their effects on clinical outcomes. *Materials and Methods*: A retrospective analysis was conducted on 258 patients admitted to the secondary-level pulmonary disease intensive care unit between January 2022 and January 2024. Patients’ demographic data, cardiological and metabolic comorbidities, laboratory parameters, and ICU-related variables were evaluated using statistical analysis methods. *Results*: The most common comorbidities were hypertension (57.0%), congestive heart failure (48.1%), diabetes mellitus (31.4%), and obesity (37.6%). Female patients had significantly higher rates of hypothyroidism, hypertension, obesity, and congestive heart failure compared to males. Patients diagnosed with chronic kidney disease (CKD) had markedly higher cardiothoracic ratios and proBNP levels. ICU length of stay was considerably longer in patients with acute kidney injury (AKI) and coronary artery disease (CAD). Cardiomegaly and obstructive sleep apnea syndrome (OSAS) were more frequently observed in obese patients. Additionally, in COPD patients, a body mass index (BMI) threshold of 25.5 was determined as a cutoff value for radiological cardiomegaly findings with a sensitivity of 69.9% and a specificity of 59.5%. Elevated pCO_2_ and bicarbonate levels in patients receiving long-term oxygen therapy (LTOT) were associated with advanced-stage COPD. *Conclusions*: Metabolic and cardiological comorbidities notably impact the clinical prognosis and ICU management of patients diagnosed with COPD and Type 2 respiratory failure. This study, which aims to provide a snapshot of the comorbidities in patients requiring ICU admission due to COPD exacerbation-related Type 2 respiratory failure but without a fatal course, seeks to highlight the key areas where preventive and protective healthcare services should be focused in this patient group. Special attention should be given to monitoring female and obese patients. Future studies should explore how individualized and preventive follow-ups and treatment approaches can improve patient outcomes, with a particular emphasis on these identified areas.

## 1. Introduction

Chronic obstructive pulmonary disease (COPD) is a progressive inflammatory disease and a major cause of morbidity and mortality worldwide. Type 2 respiratory failure associated with COPD, characterized by hypercapnia and hypoxemia, typically manifests in advanced stages of the disease and often necessitates admission to the intensive care unit (ICU). Analyzing the cardiometabolic profiles and comorbidities of this patient group is crucial for developing more effective strategies for disease management [1,2].

Cardiometabolic profiles, the interplay between respiratory functions, and comorbidities can have a direct impact on the prognosis of COPD patients. Specifically, conditions such as obesity, chronic kidney disease (CKD), acute kidney injury (AKI), diabetes mellitus (DM), hypothyroidism, and anemia can significantly influence clinical outcomes in patients with COPD-related Type 2 respiratory failure. For instance, obesity has been shown to impair respiratory muscle function and increase the need for mechanical ventilation [3,4]. Additionally, DM exacerbates inflammation levels, complicating therapeutic responses and elevating infection risks in COPD patients [5]. Hypothyroidism, by slowing metabolism and reducing energy expenditure, may lead to weight gain and obesity, both of which negatively impact respiratory function [6]. Anemia, on the other hand, compromises oxygen delivery to tissues, reducing exercise tolerance and exacerbating dyspnea, especially in COPD patients [7].

Furthermore, the coexistence of hypothyroidism and obstructive sleep apnea syndrome (OSAS) has been reported to further deteriorate respiratory function in COPD patients and heighten the risk of hypoxemia. This underscores the importance of screening for hypothyroidism in patients with concurrent COPD and OSAS [8].

The impact of these comorbidities, which accompany Type 2 respiratory failure, on ICU length of stay and responses to treatment are among the frequently studied topics in the literature. Particularly in cases of hypercapnic respiratory failure, it has been reported that hospital length of stay is significantly influenced by comorbidities and clinical parameters [9]. Furthermore, the interactions between various metabolic conditions and comorbidities clearly complicate clinical management, underscoring the need for individualized approaches and perspectives in this patient group.

In this study, we aimed to define the cardiological and metabolic comorbidities of patients who were monitored in the intensive care unit with a diagnosis of Type 2 respiratory failure due to COPD exacerbations. Our objective was to identify which metabolic profile is associated with which pulmonary or cardiological condition. The condition we aimed to present is a retrospective snapshot of the cardiometabolic profiles of COPD patients who may require intensive care.

## 2. Materials and Methods

This study was initiated after obtaining ethical approval (Approval Number: 2024-BÇEK/229 Date: 12 February 2025) from the Ethics Committee of Ankara Atatürk Sanatorium Training and Research Hospital. Patient files of all individuals admitted to the secondary-level respiratory intensive care unit between January 2022 and January 2024 were reviewed. A total of 498 patient files (admitted ICU with respiratory failure) were examined. Among these, 401 patients were diagnosed with COPD based on their pulmonary function test (PFT) results and clinical characteristics. Of these patients, 339 were diagnosed with Type 2 respiratory failure according to arterial blood gas (ABG) results and were admitted to the ICU.

**Figure 1:**
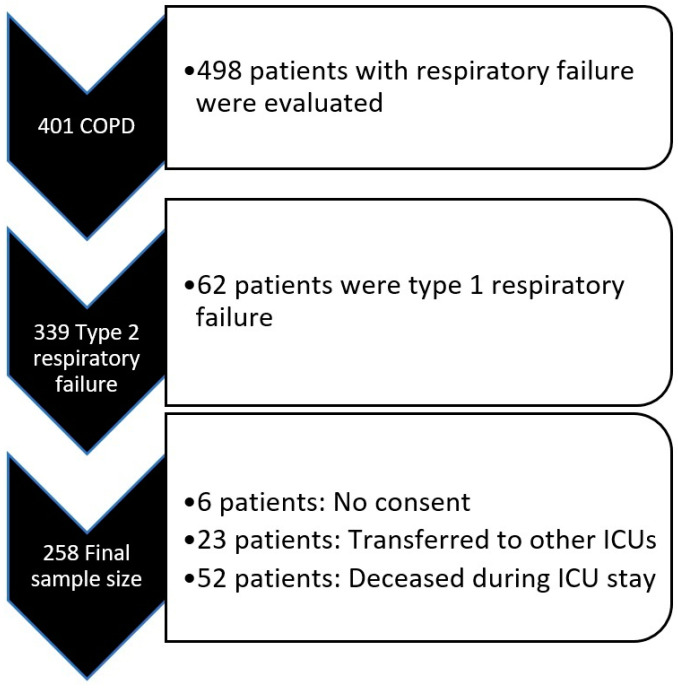
Study design. COPD: Chronic obstructive pulmonary disease.

Six patients were excluded from the study as they had left the consent box blank in the ICU admission consent form, which routinely includes a section allowing the use of medical information for research purposes. Additionally, 23 patients were excluded due to being transferred to other ICUs for various reasons. Furthermore, 52 patients were excluded as they passed away during their ICU stay. As a result, a total of 258 patients were included in the study (Figure 1).

### 2.1. Inclusion and Exclusion Criteria

All patients aged 18 years or older, diagnosed with COPD based on pulmonary function tests (PFTs) (post-bronchodilator FEV1/FVC ratio below 70% on spirometry) and clinical findings, diagnosed with Type 2 respiratory failure according to arterial blood gas analysis, and who provided consent for the use of their medical data were included in the study.

Patients were excluded if they

- Were transferred to another ICU or hospital for any reason after being admitted to the ICU;
- Passed away during their ICU stay for any reason;
- Did not provide consent for the use of their medical data as indicated on their ICU admission forms;
- Had missing data for the variables planned for analysis.

A total of 258 patients that met the inclusion criteria were enrolled in the study. Data collected included demographic information such as age, gender, and the Charlson comorbidity index (CCI), as well as the presence of metabolic, endocrinological, and cardiac diseases, particularly significant comorbidities such as diabetes mellitus (DM), hypertension (HT), hypothyroidism, chronic kidney disease (CKD), acute kidney injury (AKI), congestive heart failure (CHF), coronary artery disease (CAD), and obstructive sleep apnea syndrome (OSAS).

Patients’ body mass index (BMI) and cardiothoracic ratios (CTRs) derived from posteroanterior chest X-rays (PA-CXRs) were documented, along with the presence of cardiomegaly determined from these ratios. Laboratory parameters during ICU admission were recorded, including pH, partial carbon dioxide pressure (pCO_2_), serum bicarbonate levels, C-reactive protein (CRP), sodium, potassium, magnesium, albumin, pro-brain natriuretic peptide (proBNP), blood urea nitrogen (BUN), creatinine, T4, and thyroid-stimulating hormone (TSH).

CRP and other laboratory results obtained immediately before ICU discharge were also recorded for comparison with admission values. Additionally, whether patients required nutritional support during their ICU stay, were receiving long-term oxygen therapy (LTOT) at home prior to ICU admission, or were anemic based on hemoglobin (HGB) levels (defined as <12 g/dL in men and <11 g/dL in women) at admission was documented.

### 2.2. Statistical Analysis

Statistical analyses were conducted using IBM SPSS Statistics Version 27 (IBM Corp., Armonk, NY, USA).

The minimum number of patients to be included in the study was determined before the study commenced by utilizing the means and pooled standard deviations from similar studies. This calculation was performed using the power analysis method in SPSS software based on 80% power and a significance level of 0.05. These findings were taken into account during the data collection phase. Additionally, to account for the seasonal variability of COPD, patient data were retrospectively collected from all months of the year, ensuring that patients who were admitted to the ICU throughout the entire year were included, thereby aiming to achieve the highest possible level of homogeneity.

Categorical nominal data were presented as *n* (%). Ordinal data or numerical data that did not follow a normal distribution were presented as the median (min–max), while numerical data with a normal distribution were expressed as the mean ± standard deviation (SD).

In the patient group, for categorical variables, the chi-square test was applied when all cells contained more than 5 patients; otherwise, Fisher’s exact test was used when at least one cell contained fewer than 5 patients. Numerical data were analyzed using Student’s *t*-test if normally distributed or the Mann–Whitney U test if not. The normality of numerical data was evaluated using descriptive statistics, Kolmogorov–Smirnov and Shapiro–Wilk tests, skewness–kurtosis values, histograms, and the proximity of outliers.

For bivariate correlation analyses, Spearman’s correlation was applied if at least one of the numerical variables was non-normally distributed, while Pearson’s correlation was used if both variables were normally distributed. Effect sizes were reported using Cohen’s d value when significant differences were observed between group means for normally distributed numerical variables.

ROC (Receiver Operating Characteristic) analyses were performed, and the area under the curve (AUC) was presented along with the upper and lower confidence intervals. The cutoff values of ROC curves were reported with their respective sensitivity and specificity percentages.

For all statistical analyses, a 95% confidence interval (CI) was used, and a *p*-value of <0.05 was considered statistically significant.

## 3. Results

Of the 258 patients included in the study, 167 were male, while 90 were female. The mean age of female patients was calculated as 72.7 ± 1.3 years, while the mean age of male patients was 68.1 ± 0.69 years. Female patients were significantly older than male patients (*p*: 0.005; Cohen’s d: 0.418).

In our study, hypertension (HT) and congestive heart failure (CHF) emerged as the most common comorbidities observed in Type 2 respiratory failure associated with COPD (Table 1).

**Figure 2:**
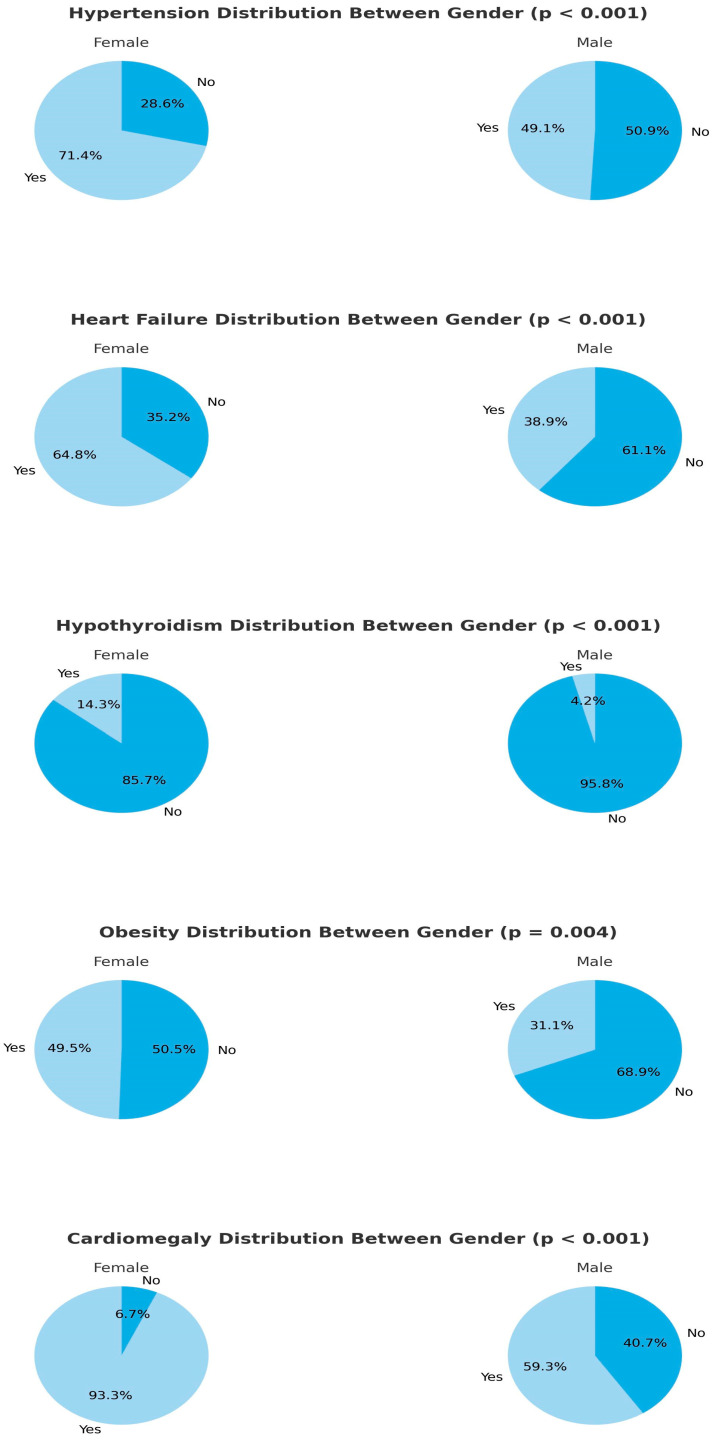
Comparison of cardiometabolic profiles between genders.

In female patients diagnosed with COPD and admitted to the ICU with Type 2 respiratory failure, the prevalence of hypothyroidism, hypertension (HT), congestive heart failure (CHF), and obesity was significantly higher compared to male patients. Additionally, the Charlson comorbidity index (CCI), body mass index (BMI), cardiothoracic ratio (CTR), blood urea nitrogen (BUN), and pro-brain natriuretic peptide (proBNP) values were significantly higher in female patients (Figure 2 and Table 2).

### 3.1. Chronic Kidney Disease and Acute Kidney Failure

Our study data showed that in COPD patients followed up in the ICU with a clinical picture of Type 2 respiratory failure, CKD was associated with a significantly higher cardiothoracic ratio compared to patients without CKD (*p* < 0.001). Moreover, the laboratory values revealed a significantly higher proBNP level at the time of ICU admission (*p* = 0.002). In addition, the incidence of cardiomegaly was significantly higher, as determined radiologically (*p* = 0.015). From the metabolic point of view, the BMI of patients with CKD was significantly higher than that of patients without CKD (*p* = 0.023). Regardless of the diagnosis of CKD, patients who were admitted to the ICU with a diagnosis of acute kidney failure in addition to COPD-Type 2 respiratory failure had a significantly longer stay in the ICU (*p* = 0.036) (Table 3).

### 3.2. Congestive Heart Failure

In patients with a diagnosis of CHF, similar to those with a diagnosis of CKD, the values of BMI were significantly higher compared to patients without a diagnosis of CHF (*p* = 0.002). In our study, the presence of CHF was also associated with the impairment of renal function. Mean values of BUN, creatinine, and potassium upon admission to the ICU were significantly higher in cases with a diagnosis of CHF as compared to those without CHF diagnosis (*p* < 0.001, *p* < 0.001, and *p* = 0.021, respectively) (Table 3).

### 3.3. Hypertension

Compared to the group without an active diagnosis of HT, the BMI values of patients with a diagnosis of HT were significantly higher (*p* < 0.001). Moreover, BUN, creatinine, and proBNP values upon ICU admission were significantly higher in HT compared to non-HT patients (for all three values: *p* < 0.001). Furthermore, in patients with HT, the cardiothoracic ratio was higher with a higher incidence of cardiomegaly and proBNP levels. (For both values, *p* < 0.001.) DM constituted the major comorbidity associated with HT (*p* < 0.001). Comparing the HT patients admitted to the ICU with non-HT patients, no significant difference could be demonstrated in admission CRP levels. However, a significant difference was noted in the CRP levels measured just before ICU discharge after treatments. Discharge CRP levels among HT patients were significantly higher compared to those without HT (*p* = 0.021) (Table 3).

### 3.4. Diabetes Mellitus

Patients with a diagnosis of DM presented with significantly higher ICU admission creatinine levels compared to those without DM (*p* = 0.003). Similarly, the incidence of AKI was significantly higher in this patient group (*p* = 0.007). Additionally, patients with DM showed a significantly higher prevalence of obesity and CAD (*p* = 0.003 and *p* < 0.001, respectively) (Table 3).

### 3.5. Hypothyroidism

In the analysis of patients with hypothyroidism as an additional comorbidity or metabolic disease, the only significant and noteworthy finding was the higher incidence of OSAS in patients with hypothyroidism (*p* = 0.001). Additionally, radiological evaluations revealed that chest diameters were smaller in patients with hypothyroidism compared to those without hypothyroidism (*p* = 0.025). This finding may be associated with the higher prevalence of hypothyroidism among female patients.

### 3.6. Coronary Artery Disease

Patients presenting to the ICU with a diagnosis of CAD had significantly higher BUN levels (*p* = 0.015). Additionally, ICU length of stay was significantly longer in patients with CAD compared to those without a CAD diagnosis (*p* = 0.024) (Table 3).

### 3.7. Anemia

As expected, patients with anemia had significantly higher CKI values (*p* < 0.001). Anemic patients were older and had lower albumin, magnesium, and potassium levels, while their TSH and proBNP levels were higher (*p* = 0.011, *p* < 0.001, *p* = 0.003, *p* = 0.037, *p* = 0.008, and *p* = 0.03, respectively) (Table 3). Similar to patients with HT, no significant difference was found in ICU admission CRP levels between anemic and non-anemic patients. However, when comparing CRP levels just before discharge, anemic patients had significantly higher CRP levels (*p* = 0.039) (Table 3). A Wilcoxon signed-rank test performed on all patients revealed that among 258 patients, CRP levels decreased in 200, increased in 49, and remained unchanged in 9 patients. Moreover, the decrease in CRP levels was significantly greater than the increase in CRP levels (*p* < 0.001). Patients with diagnoses of both HT and anemia stood out with higher discharge CRP levels compared to those with other metabolic conditions or comorbidities.

### 3.8. Obesity and Cachexia

CKI values were significantly higher in obese patients (*p* = 0.023). Additionally, these patients had higher ICU admission magnesium and creatinine levels, while their serum bicarbonate levels were lower (*p* = 0.017, *p* < 0.001, and *p* = 0.049, respectively) (Table 3). However, no significant difference was found in admission pH levels compared to non-obese patients. The incidence of cardiomegaly and OSAS was significantly higher in obese patients compared to non-obese patients. (For both results, *p* < 0.001.)

**Figure 3:**
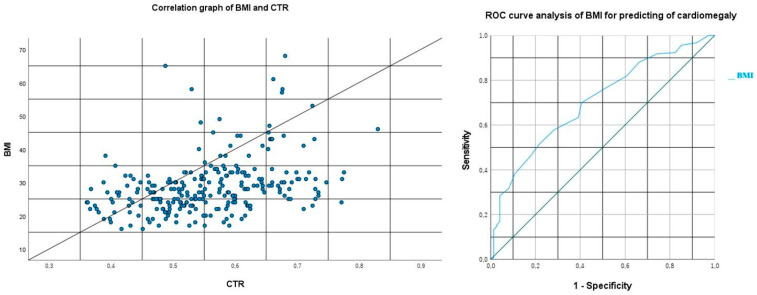
Correlation graph of the body mass index and cardiothoracic ratio. ROC curve of the body mass index for predicting cardiomegaly. BMI: body mass index, CTR: cardiothoracic ratio.

In our statistical analysis which evaluated the correlation between BMI and cardiothoracic ratio (CTR) values, a moderately positive significant correlation was identified between the two variables (Spearman: *p* < 0.001 and r = 0.404, minimum: 0.293, maximum: 0.504, and 95% confidence interval, Figure 3). Subsequently, using the ROC analysis between BMI and the radiological presence of cardiomegaly (cardiothoracic ratio > 0.5), a cutoff value of 25.5 was determined with a sensitivity of 69.9% and a specificity of 59.5% (*p* < 0.001, area under the curve (AUC): 0.704, and CI: 0.636–0.771, Figure 3).

Patients with a BMI of 18.5 or lower were classified as cachectic or at risk of cachexia. In this group, cardiothoracic ratios were significantly lower, and admission albumin levels were also reduced (*p* = 0.015 and *p* = 0.027).

### 3.9. Pleural Effusion

In our patients admitted to the ICU with COPD and Type 2 respiratory failure, the presence of pleural effusion, which was detected radiologically, at admission was found to be significantly associated with increased proBNP levels, increased CTR, and longer ICU length of stay (*p* = 0.045, *p* = 0.038, and *p* = 0.008, respectively) (Table 3).

In a multiple linear regression analysis examining factors significantly affecting ICU length of stay, including the presence of pleural effusion, AKI, and CAD, the adjusted R-squared value of the model was determined to be 0.046. This indicates that these three factors collectively explain only 4.6% of the variation in ICU length of stay, which is considered a very low explanatory value.

### 3.10. Long-Term Oxygen Therapy and Non-Invasive Mechanical Ventilation (NIMV)

Among the patients admitted to the ICU, those using LTOT at home had significantly higher pCO_2_ and bicarbonate levels at the time of ICU admission compared to non-users (*p* = 0.017 and *p* < 0.001, respectively) (Table 3).

We found that 46.5% of patients were not prescribed home NIMV upon ICU discharge, while 53.5% were prescribed NIMV. Among COPD patients with coexisting OSAS, 84.6% were prescribed home NIMV. In contrast, 51.8% of COPD patients without OSAS received NIMV. Patients with OSAS were significantly more likely to be prescribed NIMV (*p* = 0.021).

Additionally, 62.1% of COPD patients with heart failure were prescribed NIMV, compared to 45.5% of COPD patients without heart failure (*p* = 0.008).

### 3.11. Use of Corticosteroids During the Intensive Care Process

The relationships between corticosteroid use during ICU follow-ups and clinical comorbidities as well as certain laboratory parameters in patients admitted to the ICU due to Type 2 respiratory failure secondary to COPD are presented in Table 4 and Table 5. Accordingly, 70.1% of patients who received corticosteroids were male (*p* = 0.004) (Table 4). Additionally, 72.3% of patients in the non-steroid group did not have a diagnosis of diabetes mellitus (DM), suggesting that corticosteroid use was limited among patients with DM (*p* = 0.045) (Table 4).

Moreover, significant differences were observed between the groups in terms of Charlson’s comorbidity index (CCI), body mass index (BMI), CRP levels at discharge, BUN and creatinine levels at admission, cardiothoracic ratio, and white blood cell count at discharge (Table 5). Specifically, patients who did not receive corticosteroids had higher median CCI scores, higher BMI values, elevated CRP levels at discharge, higher BUN and creatinine levels at admission, increased cardiothoracic ratios, and lower white blood cell counts (Table 5).

Our patients diagnosed with COPD and admitted to the ICU with Type 2 respiratory failure presented with a variety of overlapping metabolic conditions and comorbidities. While many of our statistical analyses yielded results consistent with the medical literature, some findings were striking and noteworthy. We deemed it appropriate to summarize these findings in a table for the study’s overview (Table 6).

## 4. Discussion

This study comprehensively evaluated the effects of accompanying comorbidities and metabolic conditions on clinical outcomes in patients admitted to the ICU with COPD and Type 2 respiratory failure. Our findings largely align with the existing literature while presenting some unique and noteworthy results that contribute significantly to the clinical management of this patient group.

In our study, female patients were older and had a higher prevalence of comorbidities such as HT, CHF, obesity, and hypothyroidism compared to males, indicating a higher metabolic burden in this group. The detection of higher CTR and proBNP levels in females supports these findings. The literature also corroborates that female patients tend to have an increased burden of comorbidities with aging [10,11].

Anemic patients in our study exhibited significantly higher CCI, proBNP, and TSH levels, alongside lower albumin and magnesium levels. Although not the main focus of our study, the metabolic profile observed in anemic patients suggests the presence of anemia of chronic disease. These findings highlight the association of anemia of chronic disease with metabolic dysfunction (such as euthyroid sick syndrome) and cardiovascular burden. The existing literature reports that anemia adversely affects respiratory function and increases cardiac stress levels in chronic diseases like COPD [12]. Anker et al. emphasized that elevated proBNP levels in anemic patients are a significant marker of cardiovascular risk [13]. Additionally, the significantly higher CRP levels observed in anemic patients before discharge indicate the persistence of chronic inflammation, which can be linked to the systemic effects of anemia in COPD patients.

Chronic kidney disease (CKD) and acute kidney injury (AKI) were common comorbidities in our study, significantly influencing clinical outcomes. Increased CTR, proBNP levels, and the frequency of cardiomegaly in patients with CKD reveal the relationship between renal dysfunction and cardiovascular burden. The literature suggests that CKD is associated with left ventricular hypertrophy and cardiomegaly [14]. AKI was observed to significantly prolong ICU stay, which is consistent with prior studies showing that AKI increases mortality and morbidity in COPD patients [15].

HT is a common and impactful comorbidity in COPD patients, affecting ICU parameters. Our study found that patients with HT had higher proBNP levels, CTR, and cardiomegaly rates, indicating an increased cardiovascular risk. DM was significantly associated with obesity and AKI prevalence. The literature indicates that the coexistence of HT and DM increases the risk of cardiovascular and renal complications in COPD patients [16].

The association of pleural effusion with proBNP levels and CTR suggests that this finding may be explained by cardiac dysfunction and fluid overload. Additionally, the prolongation of ICU stays in patients with pleural effusion reflects a more complex clinical course in this group [17]. The significantly higher pCO_2_ and bicarbonate levels in LTOT users further support that these patients are in advanced stages of COPD. This may also indicate that aggressive correction of hypoxia in LTOT users could contribute to hypercarbia and metabolic compensation through respiratory center suppression due to chronic hypercarbic desensitization. A simpler explanation could be that LTOT is prescribed for more severe COPD patients. The literature also emphasizes that LTOT is associated with chronic hypoxemia and hypercapnia [18].

Obesity is a critical factor that increases the cardiovascular and metabolic burden in COPD patients. Our study found that obese patients had higher frequencies of cardiomegaly and OSAS, underscoring the importance of a multidisciplinary approach in this group. Clinical dietitians play a vital role in managing these patients. Proper nutritional management could improve both respiratory parameters and cardiovascular risk in this group. Additionally, the increased frequency of OSAS in patients with hypothyroidism may be explained by the effects of hypothyroidism on respiratory function [19].

COPD is a disease characterized by low-grade systemic inflammation. Therefore, systemic pro-inflammatory circulating markers increase the risk of cardiac comorbidities, particularly heart failure, by 2 to 5 times in COPD patients [20]. In our study, we observed that even an overweight COPD patient, despite being below the obesity threshold, is at risk for cardiomegaly. Specifically, an increased BMI in COPD patients with Type 2 respiratory failure was associated with an increased risk of cardiomegaly, with a cutoff value of 25.5. Considering that this cutoff aligns with the overweight threshold in standard classifications, we emphasize the need for strict weight monitoring during routine follow-ups to prevent increased cardiac risk in this patient group.

COPD is already known to pose a risk for cor pulmonale through various mechanisms. The additional burden of an increased BMI may further strain the cardiac reserves of these patients. Moreover, obesity itself contributes to restrictive pulmonary pathologies. Therefore, it is not surprising that an overweight COPD patient faces both an accelerated cor pulmonale development process and worsening respiratory dynamics. These findings highlight the critical role of weight control in managing COPD patients, particularly those at risk of developing cardiopulmonary complications.

The use of corticosteroids during ICU stays in patients with COPD and Type 2 respiratory failure may be associated with certain clinical outcomes. Alternatively, a clinician’s decision to initiate corticosteroid therapy may be influenced by the patient’s comorbid conditions. The findings of our study suggest that both of these scenarios may be relevant. Specifically, despite recommendations for corticosteroid use in COPD-related Type 2 respiratory failure, our results indicate that clinicians were noticeably reluctant to use corticosteroids in patients with a diagnosis of DM.

Furthermore, corticosteroid use was also lower among patients with elevated BUN and creatinine levels, increased pro-BNP levels, and higher cardiothoracic ratios. Similarly, patients with higher BMI and CCI scores were significantly less likely to receive corticosteroid therapy. These findings suggest that in such patient groups, clinicians may consider cardiometabolic comorbidities to be more relevant to the patient’s clinical status than COPD-related respiratory failure and may therefore prefer alternative treatment strategies such as diuretics or therapies aimed at reducing the cardiac load. However, it is important to note that a previous study involving 1247 ICU patients with COPD exacerbations demonstrated that systemic corticosteroid therapy improved outcomes such as mortality and the need for invasive mechanical ventilation, without a marked adverse effect profile [21,22].

Another noteworthy outcome of corticosteroid use during ICU stay in this patient population—although consistent with the known clinical effects—is the observation of lower CRP levels and higher white blood cell (WBC) counts at discharge. A secondary analysis of a randomized controlled trial on community-acquired pneumonia previously reported that corticosteroid use was associated with higher leukocyte and neutrophil counts, along with lower CRP levels [23]. Our findings are in line with these results in the literature.

While the findings of our study may contribute to intensive care management and strategies, we would like to emphasize that some of our results should not be interpreted in isolation. For instance, although BMI was frequently discussed throughout our study, we do not consider it to be a strong standalone indicator of metabolic or cardiovascular health. Both low and high BMI levels may be associated with certain metabolic and cardiac conditions; however, in patients with COPD, more comprehensive multicenter studies involving detailed analysis of pulmonary function tests, genetic and environmental risk factors, and the associated cardiometabolic comorbidities are needed to provide more robust evidence and contribute further to the literature.

### Strengths and Limitations of the Study

This study is significant for its detailed examination of the effects of comorbidities on ICU processes in COPD and Type 2 respiratory failure patients. However, the retrospective design and the single-center setting limit the generalizability of the findings.

One of the main limitations of conducting our study in a single center is the reduced generalizability of the findings. The reasons for this can be summarized as follows:

1. Limited patient diversity: a homogeneous patient population restricts the ability to compare individuals from different socioeconomic and ethnic backgrounds.
2. Lack of heterogeneity in COPD treatment and follow-up protocols: the absence of data from multiple centers eliminates potential variations in clinical practice, resulting in a uniform patient group treated according to the protocols of a single center.
3. A relatively small sample size.

## 5. Conclusions

Our study highlights the critical role of comorbidities in the clinical management of patients with COPD and Type 2 respiratory failure. Conditions such as anemia, HT, DM, CKD, CHF, and obesity necessitate closer monitoring and treatment during ICU care. Controlling systemic inflammation is particularly crucial in anemic and hypertensive patients.

This study consisted of COPD exacerbation patients with Type 2 respiratory failure requiring intensive care monitoring. It specifically focused on the cardiologic and metabolic profiles of patients who met these criteria but did not have ICU mortality (in other words, those who were not complicated due to various reasons).

To clarify further, our study population included patients who were too severe to be treated in an outpatient setting or a pulmonary ward but not critical enough to result in death in the ICU. The rationale behind this study design was to identify the cardiologic and metabolic comorbidities in COPD patients who had reached the stage of requiring ICU admission due to exacerbations. Therefore, we aimed to provide recommendations for newly diagnosed or earlier-stage COPD patients to help prevent conditions that could worsen their disease. The recommendations we have developed based on our study findings and the literature are presented in Table 7.

In this context, future studies comparing patients with ICU admissions due to COPD exacerbations and those with fewer or no exacerbations—classified under a lower GOLD stage—would be particularly valuable. This comparison would help better elucidate the impact of metabolic and cardiologic comorbidities on the risk of COPD exacerbations. All patients included in our study had an FEV1/FVC ratio below 70%. However, the degree of airflow limitation was not analyzed in detail. In future studies, patients may be classified based on their FEV1 levels, and the impact of this variable can be evaluated accordingly.

Additionally, as our study demonstrated, an increased BMI poses a radiologically assessed risk for cardiomegaly. We believe future research should focus on whether similar results would be observed in COPD patients who were not monitored in the ICU. Furthermore, it would be insightful to investigate whether these findings remain consistent when compared with the general population.

## Figures and Tables

**Table 1:** Prevalence of comorbidities in patients with Type 2 respiratory failure associated with COPD.

Comorbidity	Prevalence (%)
Hypertension (HT)	57.0
Diabetes mellitus (DM)	31.4
Coronary artery disease (CAD)	20.2
Chronic kidney disease (CKD)	17.1
Acute kidney injury (AKI)	10.9
Congestive heart failure (CHF)	48.1
Anemia	25.2
Hypothyroidism	7.8
Obesity	37.6

**Table 2:** Comparison of cardiometabolic profiles between genders.

Variable	Female Median (Min–Max)	Male Median (Min–Max)	Female Mean Rank	Male Mean Rank	*p*-Value
CCI	6 (1–12)	5 (1–12)	148.87	118.94	0.002 ^b^*
BMI (kg/m^2^)	29 (17–68)	26 (16–65)	156.99	114.52	<0.001 ^b^*
ProBNP (pg/mL)	294 (5–2590)	117 (10–2656)	151.01	117.78	<0.001 ^b^*
BUN (mg/dL)	61 (13–192)	45 (18–225)	147.37	119.76	0.005 ^b^*

BMI: body mass index, BUN: blood urea nitrogen, CCI: Charlson’s comorbidity index, and ProBNP: pro-B-type natriuretic peptide. ^b^ Man–Whitney U test. * indicates *p* < 0.05 and significant values.

**Table 3:** Comparison of clinical parameters based on different conditions.

Condition	Variable (Unit)	Present (Mean ± SD/ Median (Min–Max))	Absent (Mean ± SD/ Median (Min–Max))	*p*-Value
Chronic kidney disease	CTR	0.6 (0.37–0.73)	0.54 (0.36–0.83)	<0.001 ^a^*
	proBNP (pg/mL)	355 (22–2590)	152 (5–2656)	0.002 ^a^*
	BMI (kg/m^2^)	30 (18–53)	27 (16–68)	0.023 ^a^*
Acute kidney injury	ICU Stay Duration (days)	10 (4–367)	9 (2–66)	0.036 ^a^*
Heart failure	BMI (kg/m^2^)	29 (17–68)	26.5 (16–65)	0.002 ^a^*
	BUN (mg/dL)	60 (21–225)	40 (13–169)	<0.001 ^a^*
	Creatinine (mg/dL)	1.12 (0.4–5.7)	0.9 (0.4–3.8)	<0.001 ^a^*
	Potassium (mmol/L)	4.7 (3.2–6.8)	4.5 (2.5–7.02)	0.021 ^a^*
Hypertension (HT)	BMI (kg/m^2^)	30 (17–68)	25 (16–58)	<0.001 ^a^*
	BUN (mg/dL)	56 (13–225)	41 (16–192)	<0.001 ^a^*
	Creatinine (mg/dL)	1.1 (0.4–5.7)	0.9 (0.4–3.8)	<0.001 ^a^*
	proBNP (pg/mL)	248 (5–2656)	91 (6–2271)	<0.001 ^a^*
	CTR	0.58 (0.36–0.83)	0.51 (0.36–0.73)	<0.001 ^a^*
	CRP (mg/L)	14 (1–210)	10 (1–135)	0.021 ^a^*
Diabetes mellitus (DM)	Creatinine (mg/dL)	1.14 (0.5–5.7)	0.95 (0.4–3.8)	0.003 ^a^*
Coronary artery disease	BUN (mg/dL)	58.5 (13–192)	48.5 (16–225)	0.015 ^a^*
	ICU Stay Duration (days)	10 (3–367)	9 (2–66)	0.024 ^a^*
Anemia	CCI	6 (2–12)	5 (1–12)	<0.001 ^a^*
	Albumin (g/dL)	3.16 ± 0.06	3.58 ± 0.03	<0.001 ^b^*, Cd: 0.853
	proBNP (pg/mL)	253 (12–2497)	151 (5–2656)	0.03 ^a^*
	CRP (mg/L)	17 (1–124)	11 (1–210)	0.039 ^a^*
	Potassium (mmol/L)	4.4 (3–7.02)	4.6 (2.5–6.8)	0.037 ^a^*
	TSH (µIU/mL)	1.17 (0.11–100)	0.8 (0–13)	0.008 ^a^*
	Magnesium (mg/dL)	1.8 (1.4–2.6)	2 (1–2.7)	<0.001 ^a^*
Obesity	CCI	5 (1–12)	5 (1–12)	0.023 ^a^*
	Creatinine (mg/dL)	1.1 (0.4–5.7)	0.94 (0.4–3.03)	<0.001 ^a^*
	HCO_3_ (mmol/L)	36 (19–59)	38 (13–60)	0.049 ^a^*
	Magnesium (mg/dL)	2 (1–2.7)	1.9 (1.4–2.5)	0.017 ^a^*
Cachexia	CTR	0.49 (0.4–0.59)	0.55 (0.36–0.83)	0.015 ^a^*
	Albumin (g/dL)	3.07 ± 0.19	3.49 ± 0.03	0.027 ^b^*, Cd: 0.832
Pleural effusion	CTR	0.58 (0.36–0.73)	0.54 (0.36–0.83)	0.038 ^a^*
	proBNP (pg/mL)	253 (8–2497)	152 (5–2656)	0.045 ^a^*
	ICU Stay Duration (days)	10 (4–24)	9 (2–367)	0.038 ^a^*
LTOT	PCO_2_ (mmHg)	76 (51–132)	72 (60–126)	0.017 ^a^*
	HCO_3_ (mmol/L)	38 (13–60)	35 (21–55)	<0.001 ^a^*

SD: standard deviation; Min–Max: minimum–maximum; ICU: intensive care unit; CCI: Charlson’s comorbidity index; proBNP: *N*-terminal pro-brain natriuretic peptide; CRP: C-reactive protein; BUN: blood urea nitrogen; CTR: cardio-thoracic ratio, LTOT: long-term oxygen therapy, TSH: thyroid stimulating hormone, ^a^ Man–Whitney U Test, ^b^ Student *t*-Test, *: *p* < 0.05, significant values, and Cd: Cohen’s d.

**Table 4:** Comparison tables by corticosteroid use (nominal variables).

Clinical Variable	Condition	Corticosteroid Use (*n* = 184), *n* (%)	No Corticosteroid Use (*n* = 74), *n* (%)	*p*-Value
Hypertension	Yes	98 (53.2%)	49 (66.2%)	0.057 ^c^
	No	86 (46.8%)	25 (33.8%)	
Anemia	Yes	43 (23.4%)	22 (29.7%)	0.287 ^c^
	No	141 (76.6%)	52 (70.3%)	
Atrial fibrillation	Yes	23 (12.5%)	16 (21.6%)	0.064 ^c^
	No	161 (87.5%)	58 (78.4%)	
Obstructive sleep apnea	Yes	7 (3.8%)	6 (8.1%)	0.205 ^d^
	No	177 (96.2%)	68 (91.9%)	
Obesity	Yes	65 (35.3%)	32 (43.2%)	0.255 ^c^
	No	119 (64.7%)	42 (56.8%)	
Gender	Female	55 (29.9%)	36 (48.6%)	0.004 ^c^*
	Male	129 (70.1%)	38 (51.4%)	
Heart failure	Yes	83 (45.1%)	41 (55.4%)	0.134 ^c^
	No	101 (54.9%)	33 (44.6%)	
Pleural effusion	Yes	31 (16.8%)	20 (27.0%)	0.063 ^c^
	No	153 (83.2%)	54 (73.0%)	
Acute kidney injury	Yes	19 (10.3%)	9 (12.2%)	0.668 ^c^
	No	165 (89.7%)	65 (87.8%)	
Chronic kidney disease	Yes	29 (15.8%)	15 (20.3%)	0.384 ^c^
	No	155 (84.2%)	59 (79.7%)	
Coronary artery disease	Yes	36 (19.6%)	16 (21.6%)	0.71 ^c^
	No	148 (80.4%)	58 (78.4%)	
Diabetes mellitus	Yes	51 (27.7%)	30 (40.5%)	0.045 ^c^*
	No	133 (72.3%)	44 (59.5%)	

^c^ Pearson’s chi-square test, ^d^ Fisher’s exact test, and * significant *p* values.

**Table 5:** Comparison tables by corticosteroid use.

Variables (Unit)	No Corticosteroid Use Median (Min–Max)	Corticosteroid Use Median (Min–Max)	*p*-Value
Charlson’s comorbidity index	6 (2–11)	5 (1–12)	0.009 ^a^*
Length of hospital stay (days)	9 (2–35)	9 (2–367)	0.917 ^a^
Free T4 (ng/dL)	0.975 (0.52–2.70)	0.95 (0.40–1.90)	0.943 ^a^
TSH (uIU/mL)	1.07 (0.03–13.00)	0.81 (0.00–100.00)	0.069 ^a^
BMI (kg/m^2^)	28 (18–68)	27 (16–58)	0.004 ^a^
HCO_3_ at admission (mmol/L)	35 (13–59)	37 (19–60)	0.07 ^a^
HCO_3_ at discharge (mmol/L)	36 (20–47)	36 (14–52)	0.775 ^a^
CRP at admission (mg/L)	36 (2–433)	44.5 (1–390)	0.554 ^a^
CRP at discharge (mg/L)	20.5 (2–210)	10 (1–135)	<0.001 ^a^*
BUN at admission (mg/dL)	53 (16–225)	48 (13–169)	0.039 ^a^*
Creatinine at admission (mg/dL)	1.115 (0.50–3.03)	0.965 (0.40–5.70)	0.014 ^a^*
Cardiothoracic ratio	0.598 (0.37–0.75)	0.536 (0.36–0.83)	<0.001 ^a^*
Pro-BNP (pg/mL)	221.5 (10–2656)	150 (5–2579)	0.017 ^a^*
PCO_2_ at admission (mmHg)	73 (57–103)	76 (51–132)	0.361 ^a^
pH at admission	7.29 (7.03–7.46)	7.30 (7.04–7.47)	0.331 ^a^
PCO_2_ at discharge (mmHg)	51 (31–66)	50 (28–67)	0.219 ^a^
WBC at admission (cells/μL)	9700 (3100–29,100)	10,400 (146–24,100)	0.101 ^a^
WBC at discharge (cells/μL)	7550 (4100–16,900)	8800 (3100–26,200)	<0.001 ^a^*

ProBNP: *N*-terminal pro-brain natriuretic peptide, CRP: C-reactive protein, BUN: blood urea nitrogen, TSH: thyroid stimulating hormone, WBC: white blood cell, ^a^ Man Whitney U Test, and * significant *p* values.

**Table 6:** Summary of metabolic conditions in COPD and Type 2 respiratory failure patients.

Findings	Evidence-1	Evidence-2	Evidence-3	Evidence-4
Female patients have worse metabolic conditions.	HT (*p* < 0.001) ^a^*	CHF (*p* < 0.001) ^a^*	Hypothyroidism (*p* < 0.001) ^a^*	Obesity (*p* = 0.004) ^a^*
CKD patients are overweight and have larger hearts.	High BMI (*p* = 0.023) ^b^*	Obesity (*p* = 0.004) ^a^*	CTR (*p* < 0.001) ^b^*	Cardiomegaly (*p* = 0.015) ^a^*
AKI, CAD, and pleural effusion prolong ICU stay.	AKI (*p* = 0.036) ^b^*	CAD (*p* = 0.024) ^b^*	Pleural Effusion (*p* = 0.008) ^b^*	
CHF patients are overweight with impaired RFTs.	Obesity (*p* = 0.003) ^a^*	BUN (*p* < 0.001) ^b^*	Creatinine (*p* < 0.001) ^b^*	Admission Potassium (*p* = 0.021) ^b^*
Anemic patients are older, malnourished, and have more comorbidities and inflammation.	Age (*p* = 0.011) ^c^*	CCI (*p* < 0.001) ^b^*	Discharge CRP(*p* = 0.039) ^b^*	Low Albumin (*p* < 0.001) ^c^*
HT patients are overweight, inflamed, and have more heart and kidney dysfunctions.	High BMI (*p* < 0.001) ^b^*	Discharge CRP(*p* = 0.021) ^b^*	Cardiomegaly(*p* < 0.001) ^a^*	Creatinine (*p* < 0.001) ^b^*
DM patients are obese with renal and cardiovascular dysfunctions.	Obesity (*p* = 0.003) ^a^*	AKI (*p* = 0.007) ^a^*	Creatinine (*p* = 0.003) ^b^*	CAD (*p* < 0.001) ^a^*
LTOT users present to the hospital later.	High Bicarbonate(*p* < 0.001) ^b^*	High pCO_2_ (*p* = 0.017) ^b^*		

AKI: acute kidney injury, BMI: body mass index, CAD: coronary artery disease, CCI: Charlson’s comorbidity index, CHF: congestive heart failure, CKD: chronic kidney disease, CRP: C-reactive protein, CTR: cardiothoracic ratio, DM: diabetes mellitus, HT: hypertension, LTOT: long-term oxygen therapy, pCO_2_: partial carbon dioxide pressure, RFTs: renal function tests. ^a^: Chi-square test, ^b^: Mann–Whitney U test, ^c^: Student’s *t*-test, and *: *p* < 0.05, significant values.

**Table 7:** Recommendations for COPD patients.

**Recommendations**
√ Women at risk for COPD should prioritize weight control and attend routine check-ups. √ COPD patients should avoid nephrotoxic agents to protect their kidneys and consider renal function tests during routine follow-ups. √ Patients with kidney failure, coronary artery disease, or pleural effusion at admission should be monitored for prolonged ICU stays, and precautions should be taken to prevent potential complications. √ Anemic and/or cachectic COPD patients should collaborate with clinical dietitians to address malnutrition processes. √ COPD patients should receive brochures that emphasize the importance of sodium intake restriction and hypertension management during routine check-ups. √ Strict blood glucose regulation should be emphasized for COPD patients, and special dietary plans should be arranged for those with diabetes risk factors. √ COPD patients should be informed that pulmonary rehabilitation programs not only improve pulmonary function but also help prevent metabolic and cardiologic comorbidities.

## Data Availability

The original contributions presented in the study are included in the article, further inquiries can be directed to the corresponding authors.
